# A Comparative Kinetic and Thermodynamic Adsorption Study of Methylene Blue and Its Analogue Dye on Filter Paper

**DOI:** 10.3390/ijms26020516

**Published:** 2025-01-09

**Authors:** Andrea Bogyor, Alexandra Ana Csavdari, Tamás Lovász, Enikő Bitay

**Affiliations:** 1 Department of Chemical Engineering, Babeş-Bolyai University of Cluj-Napoca, Arany Janos Street 11, 400028 Cluj-Napoca, Romania; andrea.bogyor@ubbcluj.ro (A.B.); tamas.lovasz@ubbcluj.ro (T.L.); 2 Department of Analytical, Colloidal Chemistry and Technology of Rare Elements, Al-Farabi Kazakh National University, 71 Al-Farabi Avenue, 050040 Almaty, Kazakhstan; 3 Department of Mechanical Engineering, Faculty of Technical and Human Sciences, Sapientia Hungarian University of Transylvania, Calea Sighișoarei 2, 540485 Târgu-Mureş, Romania; bitay.eniko@eme.ro; 4 Bánki Donát Faculty of Mechanical and Safety Engineering, Óbuda University, Bécsi Street 96/b, 1034 Budapest, Hungary; 5 Research Institute of the Transylvanian Museum Society, Napoca Street 2-4, 400009 Cluj-Napoca, Romania

**Keywords:** methylene blue, methylene blue analog, adsorption, adsorption kinetics, adsorption isotherms, adsorption thermodynamics, filter paper, model quality statistical indicators

## Abstract

A comparative adsorption study was carried out for methylene blue (MB) and its 3,7-bis(N,N-(2-hydroxyethyl)amino)-phenothiazinium dye analog (MBI). Batch experiments employed aqueous solutions and commercial filter paper. Out of seven kinetic models tested by means of four quality statistical indicators, the pseudo-second-order, the double-exponential, and the bi-linear Weber–Morris equations were best fits. For both dyes, the process was described as a succession of two diffusion-controlled steps. The Freundlich isotherm was chosen from 11 models describing a variety of mechanism assumptions. Physisorption was considered responsible for the dye removal from liquid. Adsorption of MB is thermodynamically favored, whereas that of MBI is sterically hindered. Both processes are exothermic and exhibit reduced randomness at the S-L interface. The paper was found suitable for retaining MB but served rather filtration/purification purposes for MBI.

## 1. Introduction

**Scheme 1:**
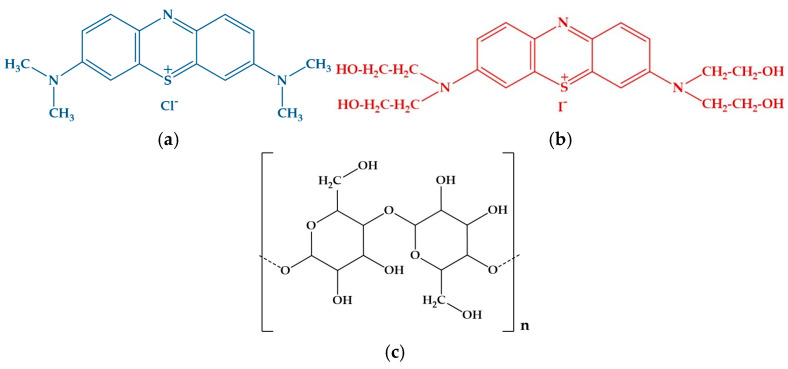
The structural formula of (**a**) methylene blue (MB); (**b**) 3,7-bis(N,N-(2-hydroxyethyl)amino)-phenothiazinium iodide (MBI); (**c**) α-cellulose. MB and MBI are coded along the entire paper in blue and red colors, respectively.

Methylene blue (MB) is one of the most widely used phenothiazine-derived dyes [1,2,3,4], either in the textile and pharmaceutical industries, or in pharmaceutical research [5]. Its molecular structure is presented in Scheme 1a. The usual research related to MB deals with methods of its removal from aqueous environments, since its concentration in wastewater may reach up to 1000 mg/L [6]. Adsorption is an environmentally friendly way of achieving this purpose [2,3], hence the ongoing quest for suitable low-cost adsorbents. Cellulose is listed among the most ecological, sustainable, and cheap adsorbents in wastewater treatment [7,8]. Its purest source derives from cotton seed hairs, since they contain between 90 and 99% cellulose [7,9]. Various other materials, such as corn husk waste [4], sugarcane bagasse [10], and all sort of biomass-derived powders [11,12,13] or biomass-generated activated carbons [6,14], have been reported.

On the other hand, an emergent trend derives from the high potential as therapeutic agents of methylene blue and its synthetic analogs [1,5,15]. MB is used as an anti-infective [16], anti-microbial, antiviral, anti-inflammatory, antioxidant, anti-tumor [1,5,15,17], and anti-psychotic active species [5]. It is a certified biological stain [18] and is thus applied in cancer treatment, research, and imaging [1,5,15,17]. Padnya et al. [5] states that the easy functionalization of the phenothiazine molecule in positions 3 and 7 opened the door for the synthesis and study of multiple MB analogs, with potential applications in photodynamic therapy, optoelectronics, or drug development. Such is the recently reported green synthesis of the 3,7-bis(N,N-(2-hydroxyethyl)amino)-phenothiazinium dye (MBI) [15]—see Scheme 1b, a larger size molecule than that of MB used here for comparison.

Potential uses of MBI have not been reported yet; although, a similar compound to both MB and MBI, 3,7-bis((2-hydroxyethyl)(methyl)amino)phenothiazin-5-ium iodide, has proved to be a photosensitizer for antimicrobial photodynamic therapy [19], as well as an oxygen colorimetric indicator for food vacuum packages [20] (the latter together with 2 other symmetrical analogs). Some (hydroxymethyl)piperidin-1-yl)phenothiazinium dyes, other novel symmetrical derivatives of MB, were reported to selectively stain the cytoplasm of tumoral OVCAR-3 and A2780 fixed cells [21]. Moreover, one of these also exhibited moderate cytotoxicity against human A2780 ovarian adenocarcinoma cells. Other MB analogs were successfully used for the cytoplasmatic staining of D407 human retinal pigment epithelial cells [22].

Hence, because of the relatively large interest of recent research towards both MB and its analogs, adsorption studies regarding these species may address either their removal from aqueous solutions, or their laboratory purification [23]. In the latter case, the adsorbent aims not to retain the dyes but rather other species that might impurify them during synthesis.

Within this context, the present study aims to describe comparatively the adsorption kinetics, isotherms, and thermodynamics of MB and MBI (see Scheme 1a and b, respectively) on a commercially available filter paper mainly constituted of *α*-cellulose [9] (see Scheme 1c). Results will show for which of the above-mentioned purposes this adsorbent is more suitable. Published research focuses mainly on the removal of MB, by characterizing either the adsorbent or the process. Furthermore, there are no literature data regarding the adsorption behavior of MBI or of any other MB analog. Hence, by taking into consideration the increasing interest in these analogs, the present study aims to fill a knowledge gap.

## 2. Results and Discussion

### 2.1. Characterization of the Adsorbent

**Figure 1:**
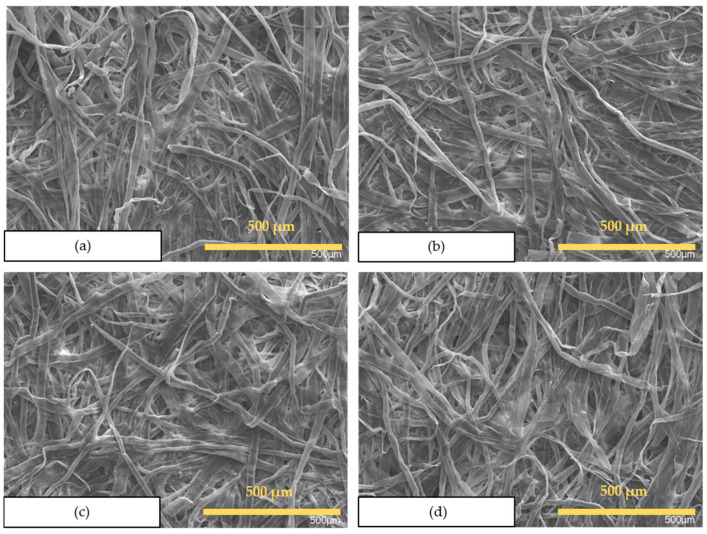
SEM images of the adsorbent filter paper: (**a**) before employment; (**b**) after 24 h when 0.05 g were kept in water; (**c**) after 24 h when 0.05 g were kept in a 5 mg/L MB aqueous solution; (**d**) after 24 h when 0.05 g were kept in a 5 mg/L MBI aqueous solution, respectively. Temperature was kept constant at 298 K.

The filter paper used as the adsorbent is described by the manufacturer’s documentation [9] as product MN 751. Its characteristics below justify its choice:

(i) It is obtained from the refined pulp of short-fibred seed hairs from cotton seeds, a low-cost, renewable, and environmentally friendly source;
(ii) It is highly standardized (see Figure 1a);
(iii) It consists of 95% *α*-cellulose;
(iv) It is virtually ashless, with an average content of about 0.1% ash. Hence, the provider guarantees that no chemical species will be extracted or leaked from the paper during employment;
(v) It is recommended for general laboratory use, in most cases as an alternative to another product with good absorptive capacity (MN 713).

The manufacturer [9] does not mention the paper’s adsorptive capacity. Hence, its BET surface was measured. The obtained value, 0.7992 ± 0.0260 m^2^/g, is approximately half of the one measured for pure cellulose [24], but relatively low when compared with other readily available adsorbents, for example, un-activated charcoal [25], or some recently described activated carbon varieties [6,14].

Figure 1 presents SEM images recorded before and after the adsorbent’s employment. Figure 1a corresponds to the dry paper, before experiments. Figure 1b–d illustrate the adsorbent after adsorption equilibrium was reached in water, MB, and MBI aqueous solutions, respectively. Prior to SEM records, the paper flakes were dried for 24 h at 303 K. The images prove that no structural damage was caused to the adsorbent during the process. The paper only changed color, turning blue in case of MB, and very light blue in case of MBI, respectively. However, this fact cannot be sensed via SEM images.

The images in Figure 1 show that the paper is constituted of multiple/tangled layers of cellulose fibers, with fiber thickness varying randomly between approximately 4 and 35 μm. It is obvious that this is not a particulate microporous structure, instead it exhibits interstitial spaces of various dimensions, which ought to permit any liquid to flow trough. This is also why the BET analysis could not produce a micropore area, nor a micropore surface for this adsorbent.

On the other hand, since this filter paper has been manufactured primarily for the purpose of ensuring a certain filtration speed, it still might have a high porosity, thus permitting big size molecules like MBI to enter its inner structure. For example, the review of Hokkanen et al. [7] mentions pore diameters of 1.93 nm for unmodified and of 20.41 nm for pure cellulose, respectively.

### 2.2. Principle of Experiments

**Figure 2:**
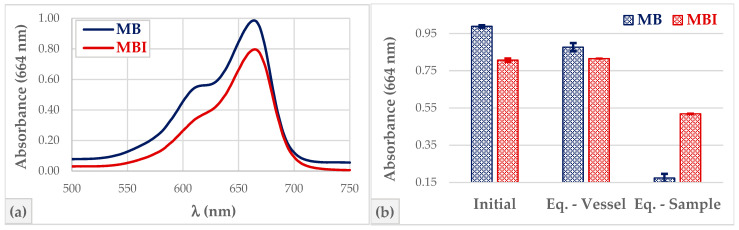
(**a**) Visible range molecular absorbance spectra for 5 mg/L aqueous solutions of MB and MBI, respectively; (**b**) Initial and equilibrium 664 nm absorbance values for 5 mg/L dye solutions. “Eq.-vessel” data are recorded for the bulk liquid after 24 h without the paper adsorbent, whereas “Eq.-sample” correspond to experiments involving 0.05 g paper. Data were obtained at 298 K.

Figure 2a illustrates part of the visible spectra of the two dyes, each for a 5 mg/L aqueous solution. It is obvious that MB and MBI have similar molecular absorbance spectra, and both exhibit a maximum at 664 nm [1,15]. The calibration lines obtained at this wavelength are described by Equations (1) and (2), respectively, in which the concentration values C_MB_ and C_MBI_ are expressed in mg/L. Hence, the molar absorptivity of MBI, of 82,900 ± 1063 L/mole·cm, is reported here in premiere.

A_664_ = (5.78 ± 1.30) × 10^−2^ + (1.88 ± 0.03) × 10^−1^ C_MB_ (1)

A_664_ = (0.13 ± 1.02) × 10^−2^ + (1.56 ± 0.02) × 10^−1^ C_MBI_ (2)

According to the above findings, the extent of adsorption for both methylene blue (MB) and 3,7-bis(N,N-(2-hydroxyethyl)amino)-phenothiazinium iodide (MBI) could be assessed by means of the absorbance values of the bulk liquid. These are related to the remanent dye concentrations by means of calibration (Equations (1) and (2)).

**Figure 3:**
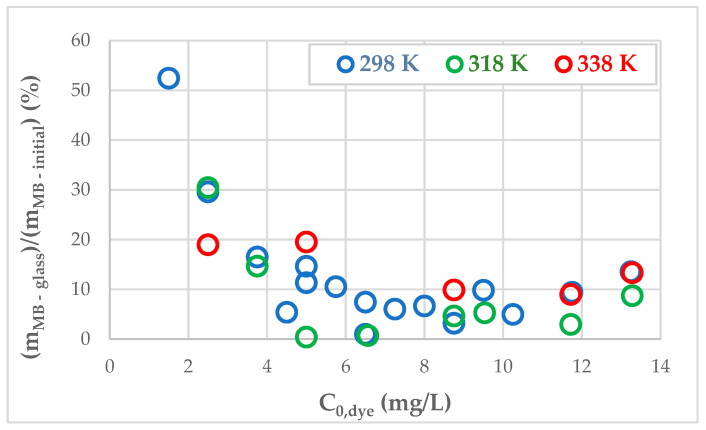
Mass percentage of MB retained by the glass recipient’s inner surface at 24 h from process initiation, as compared to its initial mass in the liquid phase, vs. the initial mass ratio adsorbate (dye)/adsorbent (paper), at 298, 318 and 338 K, respectively.

Figure 2b illustrates comparatively, see columns marked “initial” and “Eq.-sample”, how the 664 nm absorbance values drop for both MB and MBI when the process reaches equilibrium. The differences in the A_664_ values correspond to the amounts of dye retained by solid adsorbents in contact with the liquid. In the case of MB, these are both the paper and the inner surface of the employed glass vessels. The fact that the glass also retains MB is demonstrated by the smaller value of A_664_ in the “Eq.-vessel” column, which describes experiments carried out in the absence of paper, as compared to the corresponding, approximately 13% higher “initial” value (see Figure 3). On the other hand, MBI absorbance remains unaffected; hence, this species is not or is insignificantly adsorbed by the glass recipient’s inner walls. This finding leads to the introduction of an additional sets of experiments to be carried out just for MB, and without paper. Their results were used to differentiate between the adsorptive capacities of paper and glass, as well as to calculate the correct values for paper only.

Figure 3 presents on its *Y*-axis the glass retained MB mass percentage out of the total initial mass in the sample. The *X*-axis stands for the initial dye concentration in the liquid. Data were recorded at equilibrium (24 h after process initiation), at all three employed temperatures. It can be observed that at lower initial dye concentrations, the adsorbent solids (the recipient’s glass inner walls and the filter paper, respectively) compete strongly for the adsorption of MB. At C_0,MB_ of 1.5 mg/L, for example, approximately half is retained by the vessel. Yet, as the C_0,dye_ increases, MB adsorption by paper is more favored, so that after a certain threshold, no more than 10% is lost to glass. Temperature seems to have no significant influence on this tendency, since respective experimental data presented in Figure 3 almost superpose.

Absorbance measurements, carried out both in the presence and in the absence of filter paper, permitted calculus of remanent/un-adsorbed dye concentrations, as well as of the paper’s adsorption capacities q (mg/g)—see Equation (3). At process equilibrium, q reaches its maximum value q_e_ (mg/g).

(3) $$q=\frac{\left(C_{0,dye}-C_{dye}\right)V_{sample}}{m_{paper}}$$

C_0,dye_ and C_dye_ stand for the initial and momentary concentrations of MB or MBI, respectively, and are expressed in mg/L. In the case of MB, C_dye_ is a remanent dye concentration caused solely by filter paper adsorption. It is calculated from absorbance differences such as the one between columns “Eq.-vessel” and “Eq.-sample” in Figure 2b. Hence, it is a corrected value which eliminates glass-adsorbed MB. No such corrections were necessary for MBI. In Equation (3), both V_sample_, the volume of the bulk liquid (aqueous dye solution), and m_paper_, the mass of employed adsorbent paper, have the same values in each experiment; these are 5 mL and 0.05 g, respectively.

**Figure 4:**
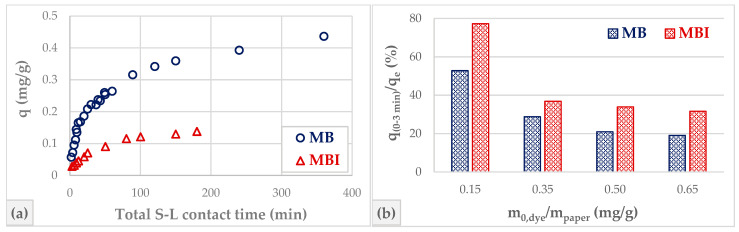
(**a**) Typical kinetic curves q vs. total S-L contact time, for m_0,dye_/m_paper_ of 0.5 mg/g and 298 K; (**b**) process advance towards equilibrium, expressed as % achieved out of q_e_ within the first 3 min after initiation, at 298 K.

Kinetic experiments recorded q vs. total S-L contact time curves, such as those presented in Figure 4a. On the other hand, equilibrium data consisted of q_e_ vs. C_e_ pairs, like those in Section 2.5.1, where C_e_ (mg/L) stands for the dye bulk concentration (C_dye_) at equilibrium.

### 2.3. Statistical Parameters Used in Kinetic and Thermodynamic Model Quality Assessment

One goal of this work is to describe comparatively the kinetic and isotherm models which best fit the adsorption process of MB and MBI on the employed filter paper. To differentiate between the prediction quality of these models, as well as to decide in favor of one of them, a set of statistical parameters were employed [26,27,28]. None of these proves by itself which model is more satisfactory; hence, information given by all together was considered for a better model quality assessment.

Appendix A describes the indicators employed in this work and motivates their choice. These were the adjusted coefficient of determination (R^2^_adj_), the root means square error (RMSE), and the hybrid fractional error deviation (Hybrid), respectively. Additionally, error distribution of residuals helped identification of models with undesired error trends.

All statistical indicators were employed for the assessment of kinetic model quality, whereas isotherm models were analyzed only from the perspective of R^2^_adj_ and Hybrid values.

### 2.4. Kinetic Data and Models

#### 2.4.1. Kinetic Data

Kinetic adsorption data were gathered for both MB and MBI at four different initial dye/paper mass ratios: 0.15, 0.35, 0.5, and 0.65 mg/g, respectively. The first q vs. t experimental point was usually obtained at 2–3 min after process initiation, whereas the dye’s partition equilibrium between liquid and solid phases was reached after 24 h. Experiments were carried out at constant temperatures of 298, 318, and 338 K, respectively.

Figure 4a depicts typical paper adsorptive capacity q vs. total S-L contact time, for a 0.5 mg/g m_0,dye_/m_paper_ ratio, at 298 K. MBI is retained less than MB, probably because it is sterically hindered by its polar functional groups to bond to the polar *α*-cellulose chain (see Section 2.5.3). MB adsorption is somewhat faster, yet for both MB and MBI, the overall process slows down as the adsorbent’s surface coverage nears saturation. The first experimental q values are well above zero, suggesting that the process has already advanced within its first 3 min.

Figure 4b shows how much of the equilibrium value q_e_ has been reached within this time frame, for both MB and MBI, at 298 K and various m_0,dye_/m_paper_ ratios. When drawing conclusions, the results in Figure 4b may be corelated with those in Figure 3. Hence:

(i) The higher the initial dye concentration of the liquid, the less of the process advances within these first minutes,
(ii) The fast initial process advance for 0.15 mg/g m_0,dye_/m_paper_, corresponding to C_0,dye_ of 1.5 mg/L, is due in the case of MB to adsorption both by the paper and the vessel’s inner surface (see the significant corresponding values in Figure 3). In the case of MBI, it is due solely to paper adsorption.

Data collected at higher temperatures proved that the values in Figure 4b were just slightly affected; they dropped with as little as 3% within a 40 degrees range.

Whatever the case, proper kinetic models can be put forward only if at least three successive half-lives, meaning 87.5% of process advance until equilibrium, is described by experimental data. Therefore, the kinetic models proposed here are discussed for the 0.5 mg/g m_0,dye_/m_paper_ curves, for which approximately 80% of process advance is covered by data for MB, and 67% for MBI, respectively (at 298 K).

#### 2.4.2. Employed Kinetic Models

Table 1 summarizes the seven adsorption kinetic models [27,28,29,30,31,32,33,34] against which data such as those in Figure 4a were tested. They are either empirical or derived from some assumptions regarding the adsorption mechanism.

Among the first category, the pseudo-first-order (PFO), the pseudo-second-order (PSO), and the Avrami “fractal like kinetic” models are often reported for methylene blue and other dyes [28,29]. PFO and PSO rate laws assume first- and second-order increase in q over time, with constant rate coefficients for the entire process duration. PFO and PSO are typically connected to physical adsorption and chemisorption, respectively [6]. On the other hand, the Avrami model assumes a time-dependent rate coefficient, with its effective value equaling (n*k_Avrami_*t^n−1^) [28] (See Table 1).

**Figure 5:**
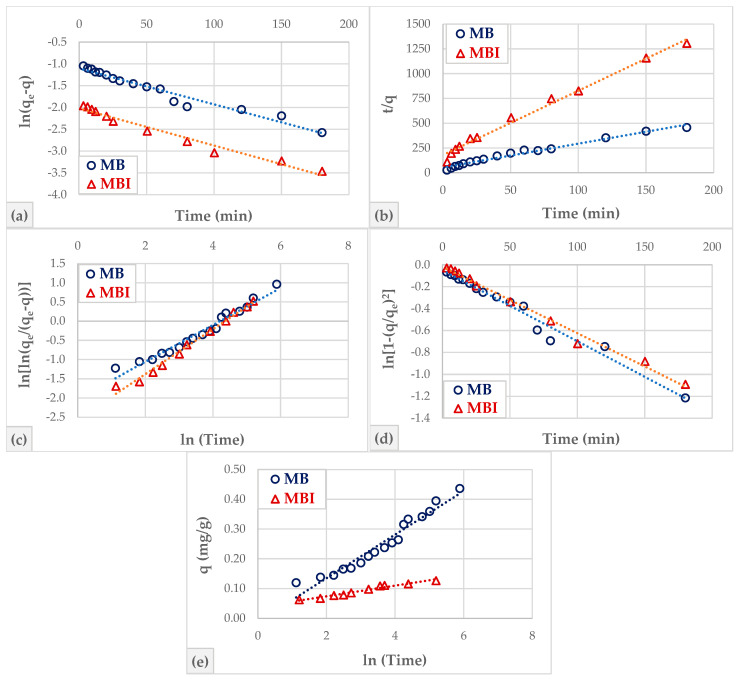
Plots of linearized 2-parameter rate laws for both MB and MBI, in case of: (**a**) the PFO model; (**b**) the PSO model; (**c**) the Avrami model; (**d**) the Vermeulen model; (**e**) the Elovich model. Experimental data correspond to m_0,dye_/m_paper_ of 0.5 mg/g and 298 K.

PFO, PSO, and Avrami plots of corresponding Equations in Table 1 are presented for both dyes in Figure 5a–c, respectively. These show that good lines can be obtained, which permit the calculus of k_PFO_, k_PSO_, k_Avrami_, and n. Calculated values of q_e_ may also be obtained and compared with their experimental correspondents. This constitutes another criterion for kinetic model suitability. Further quality assessment of these models is discussed in Section 2.4.3 and Section 2.4.4.

Among another category of rate laws are those describing an intraparticle diffusion-controlled process: the Vermeulen (also known as “pore–surface mass diffusion”) and the Weber–Morris [28,32] models, respectively. The Vermeulen equation in Table 1 is used to check whether the intraparticle diffusion (IPD) is the sole rate determining process. If so, a straight line passing through the origin ought to be generated by its plots, with the slope equaling k_Vermeulen_. The latter ought to depend linearly on each species’ intraparticle diffusivity. Vermeulen plots are shown in Figure 5d for MB and MBI, respectively. Their good linearity and intercept near to zero proves that IPD seems to be the rate-determining step for the adsorption of both dyes. On the other hand, the adsorbent is not a particulate matter, so that IPD refers here to transport phenomena inside the spaces formed by the multiple fiber layers—see Figure 1. Further quality analysis of this model is presented in Section 2.4.3.

The Weber–Morris (WM) rate law, often referred to as the “IPD model”, is one of the most frequent ones used to describe S-L adsorption kinetics [28,29,30,31,32]. The slope of its equation in Table 1 equals the rate coefficient k_WM_, whereas its intercept B has been assigned multiple physical meanings. Tan et al. [28]. consider that it stands for an initial adsorption rate. Others regard it as some measure of the S-L boundary layer thickness, hence explaining its potentially high value by means of large film diffusion resistance [30].

**Figure 6:**
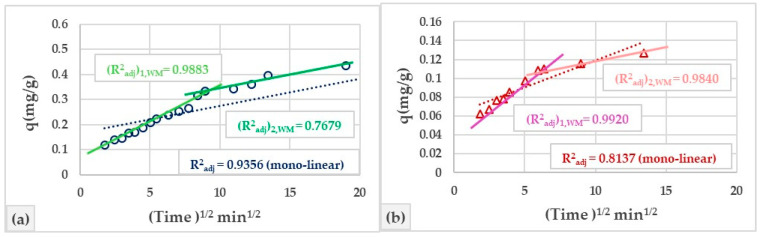
Plots of the 2-parameter Weber–Morris rate law for both the mono- and the bi-linear approaches, in case of (**a**) MB; (**b**) MBI. Experimental data correspond to m_0,dye_/m_paper_ of 0.5 mg/g and 298 K.

WM equation plots are presented in Figure 6a,b for MB and MBI, respectively. It is obvious that piecewise linearization, such as a sequence of 2 successive lines with decreasing slopes, describes the plot $\left(q\;vs.\;\sqrt{t}\right)$ better than a single line. WB multilinearity is explained by multiple mechanisms simultaneously controlling the rate [31], such as IPD and film diffusion, respectively. Each individual segment may be assigned to a certain time frame and therefore corresponds to one dominant, or several simultaneous rate controlling mechanisms. This model will be analyzed from both the perspectives of the mono- and bi-linear plots in Section 2.4.3 and Section 2.4.4.

A kinetic law describing far from equilibrium chemisorption on highly heterogeneous adsorbents is the Elovich model [28,34] (see Table 1), in which α stands for the initial adsorption rate, whereas β for a constant related to the extent of surface coverage [28,34]. Plots of its equation are presented in Figure 5e. The suitability of this model for both MB and MBI is discussed in Section 2.4.3 and Section 2.4.4.

All six rate laws listed above are 2-parameter models, thus permit linearization (see Table 1). However, multiple parameter models were also proposed for better description of experimental data [28,32]. Within this context, the 4-parameter double exponential kinetic law (DE) in Table 1 has been considered. Although being an empirical model, it offers some hints related to the process mechanism, by suggesting more than one path for the adsorbate’s uptake.

In this case, two parallelly occurring steps are assumed, both of first order, but with rate coefficients which have different orders of magnitude [28,32,33]. The process begins with rapid external as well as internal diffusion. These control the overall rate and are characterized by the overall k_1,DE_ (1/min) rate constant. As the process advances and the immediate available adsorbent surface approaches saturation, the slower transport in the interstitial spaces of the paper’s fiber net takes over (see Figure 1), eventually controlling the overall rate. This stage is described by k_2,DE_ (1/min), and its values are usually lower than those of k_2,DE_. The model constants A_1_ and A_2_ contain information about the diffusivity of each species and can lead back to a calculated q_e_ value.

#### 2.4.3. Analysis of Kinetic Model Quality and Choice of Most Suitable Model(s)

As it may be observed from Figure 5 and Figure 6, almost all 2-parameter rate laws could potentially fit experimental data, maybe for MBI more so than for MB. Hence, these were compared by means of the model quality statistical indicators R^2^_adj_, RMSE, and Hybrid (see Section 2.3 and Appendix A). Their values for MB are presented in Appendix B.

The most suitable kinetic models were identified first for MB. Only then were these employed for the processing of time-resolved data of MBI, so that process dynamics of the two dyes might be compared. Therefore, Appendix B contains only MB values of employed model quality indicators.

**Figure 7:**
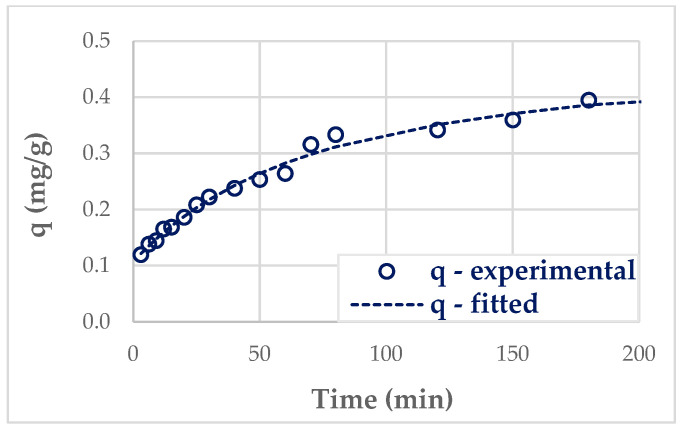
Experimental and DE model predicted kinetic data for MB, at m_0,dye_/m_paper_ of 0.5 mg/g and 298 K.

Kinetic parameters for rate laws defined by linear equations in Table 1 were determined from their corresponding line intercepts and slopes, whereas those of the 4-parameter equation by means of curve fitting tools. Figure 7 illustrates the good fit between the calculated experimental and model values for the same MB data as presented in Figure 5 and Figure 6.

Data in Table A1 (see Appendix B) ought to be analyzed from the simultaneous perspective of R^2^_adj_ desired value close to 1, as well as desired RMSE and Hybrid values close to 0. Because of high Hybrid function values, the PFO, Avrami, and mono-linear Weber–Morris kinetic models could be ruled out. The latter requires a multi-linear approach. The remaining PSO, Vermeulen, Elovich, and DE rate laws showed comparable results. Additionally, the good statistical indicators of the Vermeulen model suggested that in all employed experimental conditions, the adsorption of MB was mainly intraparticle diffusion controlled and further supported the necessity of at least bi-linear fits of the WM rate law (see also Figure 6).

**Figure 8:**
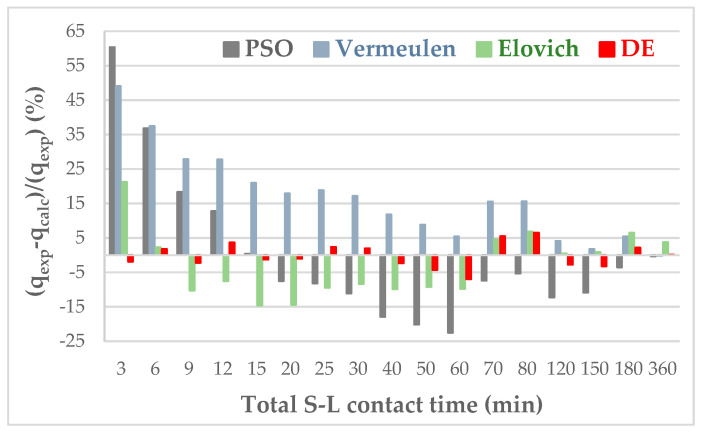
Kinetic model error distribution over process course (total S-L contact time) for the adsorption of MB, for m_0,dye_/m_paper_ of 0.5 mg/g and 298 K.

For further differentiation among the remaining models, their time-resolved error distribution (see Equation (A4) in Appendix A) has been plotted in Figure 8. The good quality and reproducibility of experimental data are proven by the fact that triplicate measurements had standard relative deviation values lower than 4% (see Section 3). Hence, the data presented in Figure 8 truly reflect kinetic model quality as well as their corresponding errors.

The positive errors given by the Vermeulen model showed that it predicted smaller than experimental q values, for the entire course of the process. Hence, it was ruled out.

The PSO, Elovich, and DE models gave both positive and negative errors, with much smaller and better distributed values for DE. Hence, the DE model was retained. Its good performances are due to its four parameters, hence to a more suitable description of experimental data range.

The positive errors of over 35% given by the PSO rate law during the first 6 min are because it describes the entire course of adsorption by means of kinetic parameters calculated from data collected after already approximately 20% process advance has been completed (see Figure 4b). On the other hand, negative errors of over 20% within the mid time range of the process might be explained by PSO being a simple 2-parameter empirical model, which does not describe the adsorption mechanism, and hence, has poor prediction capacity. However, it was not completely ruled out since it is widely reported by the literature for MB [28,29].

Errors of the Elovich model exhibited a similar distribution as those of the PSO yet had considerably smaller values. However, some values are as high as 15%, probably due to the following:

(i) the process being described again by a simple 2-parameter model, with α and β values being calculated from data describing at most the “remaining” 80% of process advance towards equilibrium, and
(ii) the process not being a chemisorption (see Section 2.5.1 and Section 2.5.3) and not far from equilibrium, hence not following the model assumptions.

However, this rate law was also not disregarded because it manages to give some information related to the initial rates, by means of α values presented in Table 2.

In conclusion, the PSO, Elovich, and DE kinetic models were found suitable to describe the advance of MB adsorption on filter paper, with the remark that the WB rate law requires a bi-linear approach (see Figure 6). Therefore, these equations were applied for processing all time-resolved data for both MB and MBI, with the final purpose of comparing the adsorption dynamics of these two dyes.

#### 2.4.4. Process Kinetics and Kinetic Parameter Values

Table 2 contains kinetic parameters for both MB and MBI, calculated according to the PSO and Elovich rate laws, respectively. Results corresponding to 1.5 mg/g initial dye/paper mass ratio are not given, since they are not relevant. In this particular case, the process advances more than 50% for MB and almost 80% for MBI, within the time frame necessary to record the first experimental q vs. t point (see Figure 4b). Hence, calculated kinetic parameters would only describe the process in the vicinity of equilibrium. This is also true for the other kinetic models.

Pseudo-second-order rate coefficients k_PSO_ decrease with rising m_0,dye_/m_paper_ ratios, even though the initial dye concentration gradient between liquid and solid is higher. This demonstrates that the overall process is most probably diffusion controlled; the higher the dye concentration in the liquid, the faster the outer adsorbent surface saturation, hence the more time molecules need to reach the still available surface inside the spaces of the paper’s fiber net.

On the other hand, increased temperature speeds up both MB and MBI removal from the liquid phase. The apparent activation energies were calculated by means of Arrhenius linearization. Values of 30.74 and 25.12 KJ/mole were obtained for MB and MBI, respectively, both values sustaining a diffusion-controlled process [35]. The value for MBI is surprisingly smaller than that for MB, despite its bigger molecular size. Yet, let us not forget that the PSO model is not the best one to describe dynamic behavior (see the error distribution in Figure 8), so that its apparent process activation energies ought to be regarded with reserve.

In case of the Elovich rate law, α is a measure of the initial adsorption rate. According to previously reported findings, it increases with increasing initial dye concentrations, as well as with temperature [28,34]. Data in Table 2 are not relevant in describing C_0,dye_ dependence, neither for MB nor MBI, because of too few experimental points within a narrow range of values. Yet, they enable the following apparent activation energies: 23.14 ± 3.84 for MB (with 0.9464 R^2^_adj_) and 1.40 ± 0.03 KJ/mole for MBI (with 0.9991 R^2^_adj_), respectively.

The value for MB is somewhat smaller than its equivalent derived from the PSO model, but of the same order of magnitude, whereas the value for MBI is significantly lower. Moreover, temperature dependence of equilibrium data (see Section 2.5) suggests physisorption, in disagreement with the chemisorption assumption of the model. Additionally, even though the adsorbent is constituted of multiple, net-like, cellulose fiber layers, it might have otherwise highly homogeneous proprieties, since it is manufactured by a strictly controlled procedure, which ensures tight quality control of the final product. Hence, the strong heterogeneous adsorbent surface assumption of the model might not be met.

The values of β are assigned the significance of a “desorption constant related to the extent of surface coverage and activation energy for chemisorption” [28,34]. It was reported that β increases with increasing C_0,dye_ and decreases with rising temperatures [28]. Data in Table 2 prove the first dependence to be the opposite, higher dye load in the liquid resulting in less surface coverage. On the other hand, β values in Table 2 are irrelevant regarding the effect of temperature, since no positive or negative evolution trend could be identified.

**Figure 9:**
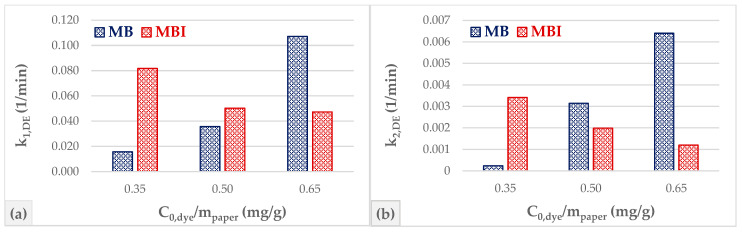

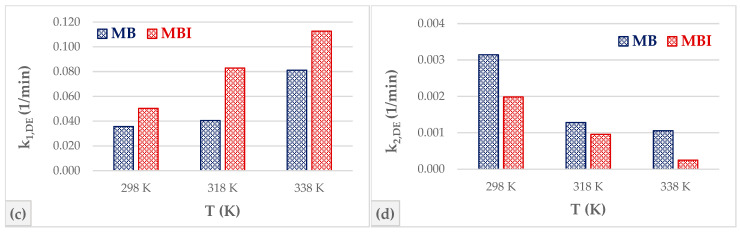
Average values of first-order rate coefficients corresponding to the DE kinetic law: dependence on initial dye/paper mass ratio, at 298 K, for (**a**) k_1,DB_ and (**b**) k_2,DB_, respectively; dependence on temperature at m_0,dye_/m_paper_ of 0.5 mg/g, for (**c**) k_1,DB_ and (**d**) k_1,DB_, respectively.

The double exponential kinetic model involves two individual first-order rate coefficients. k_1,DB_ describes the fast increase in q at the beginning of the process (see Figure 4a), whereas k_2,DB_ corresponds to the slower portion, closer to equilibrium. Their values are presented in Figure 9 for both dyes, as a function of initial adsorbate/adsorbent mass ratio (Figure 9a,b) and temperature (Figure 9c,d), respectively. Regardless of the experimental conditions, the slower stage exhibits one order of magnitude lower rate constants. Otherwise, values for MB and MBI are comparable in every analyzed category.

The results exhibit well-defined trends: both k_1,DB_ and k_2_,_DB_ increase with increasing initial liquid phase dye concentration in case of MB, but decrease in case of MBI. For methylene blue, this fact can be explained by the enhanced concentration gradient between the liquid bulk and the adsorbent’s surface. This is the driving force of diffusion/transport of MB molecules towards the paper’s surface as well as inside its interstitial spaces, hence the higher rate coefficients. On the other hand, even though the same drive could potentially act for MBI, it seems that its considerably larger molecular size does not allow its penetration into the adsorbent’s spaces and/or encounters large film diffusion resistance. It could also cover as much as possible the immediate and readily accessible outer surface of the paper, saturating it, hence blocking further adsorption. As a result, the process slows down. However, the explanation might just be relying on the steric hindrance of MBI adsorption, which increases together with its concentration in the liquid phase (see Section 2.5.3).

Figure 9c,d illustrate the effect of temperature on both k_1,DB_ and k_2_,_DB_. With its increasing value, the rapid stages speed up, whereas the slow stages slow down, for both MB and MBI, respectively. The latter is probably due to enhanced film diffusion resistance within the interstitial cavities of the paper’s fiber layers (see also the discussion on the bi-linear Weber–Morris model).

Results presented in Figure 9c enabled the calculus of apparent activation energies for the faster process stage. Obtained values were alike, of 16.32 and 16.36 KJ/mole for MB and MBI, respectively. These indicate that the process is diffusion-controlled [26]. The k_2,DE_ constant’s decrease in Figure 9d can be described by a similar to Arrhenius dependence but with negative apparent activation energies. These do not have any physical meaning; they just express the extent of temperature effect on k_2,DE_. Strongly different values were obtained for MB and MBI, of −22.27 and −41.91 KJ/mole, respectively. Hence, the slow IPD and film diffusion-controlled stage of MBI is more impeded by increasing temperatures than that of MB.

**Figure 10:**
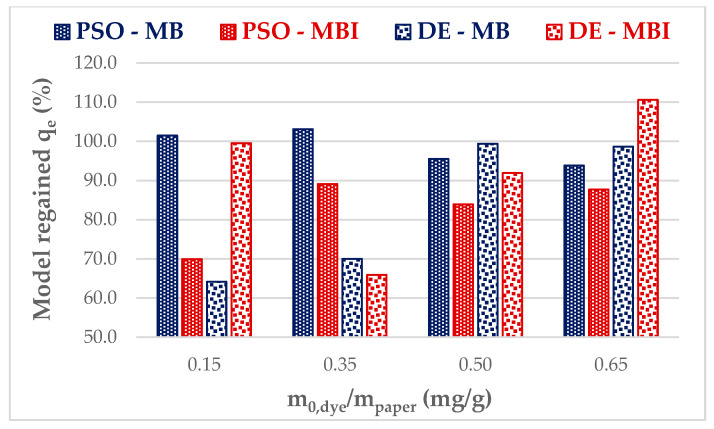
Percentage of kinetic model regained average q_e_ values, as compared to their respective average experimental correspondents, at 298 K.

Both PSO and DE kinetic models permit assessment of q_e_ from their respective time-resolved data. Hence, comparison between the model-predicted and experimental q_e_ values might be regarded as a supplementary decision criterion in favor of one or another model. Figure 10 presents regained percentages of experimental q_e_ values by means of kinetic model prediction, for both MB and MBI, as a function of initial adsorbate/adsorbent mass ratio. Suitable kinetic laws ought to generate values close to 100%.

The PSO rate law predicts well q_e_, at least for MB, regardless of m_0,dye_/m_paper_, yet could do better when describing the time course of adsorption (see Figure 8). On the other hand, the DE model calculates appropriate q_e_ only at higher dye loads but performs well in process advance description.

**Figure 11:**
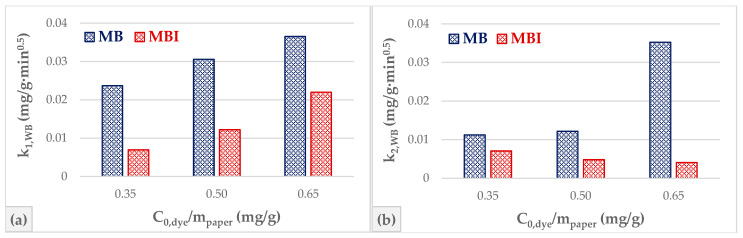

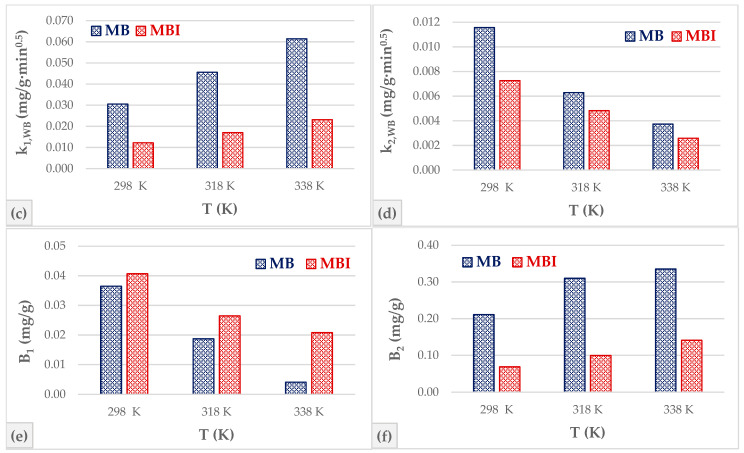
Average values of the Weber–Morris kinetic parameters for its bi-linear approach. Diffusion-controlled rate coefficients dependence on initial dye/paper mass ratio, at 298 K, for (**a**) k_1,WM_ and (**b**) k_2,WM_; as well as on temperature, at m_0,dye_/m_paper_ of 0.5 mg/g, for (**c**) k_1,WM_ and (**d**) k_2,WM_, respectively. Model constant dependence on temperature, at m_0,dye_/m_paper_ of 0.5 mg/g, for (**e**) B_1_ and (**f**) B_2_, respectively.

Figure 6 proves that a bi-linear approach of the Weber–Morris kinetic law is more suitable for the description of MB and MBI adsorption. It shows that the “pore” diffusion rate constant, given by the slope of each linear segment, decreases in two successive steps. This is due to the diminishing role of IPD and the increasing role of film diffusion [28,30,31], respectively. Hence, differentiation can be made between the faster transport in the larger inner spaces of the adsorbent cellulose fiber net (see Figure 1) and the slower transport in the smaller ones, respectively. Corresponding kinetic parameters are rate coefficients k_1,WM_ and k_2,WM_, as well as model constants B_1_ and B_2_, respectively. The latter are related to S-L boundary layer thickness and are thus indicators of high film diffusion resistance [30]. The results are presented in Table 3 and Figure 11 for both dyes.

Total S-L contact time frames allocated for each linear segment of the WM bi-linear approach could be identified clearly in the respective plots. For both MB and MBI, the first stage was between 3 and 50 min, with as low as 3 to 25 min intervals at higher temperatures, and the second stage, also depending on temperature, between 30 and 50 to 170 and 250 min.

The kinetic parameters presented in Figure 11 resemble results obtained by the DE kinetic model (see Figure 9), in the sense that:

(i) Obtained values have the same order of magnitude for both dyes.
(ii) At the same temperature, k_1,WM_ values are higher than k_2,WM_ ones (Figure 11a–d resemble Figure 9a–d). This fact is supported by results given in Table 3, which exhibit lower B_1_ than B_2_ values. Since these are associated with S-L boundary layer thickness and film layer diffusion resistance, their evolution with varying experimental conditions ought to reflect the reverse tendency as followed by their corresponding k_WM_ values.
(iii) For both dyes, k_1,WM_ increases with increasing m_0,dye_/m_paper_ (see Figure 11a). The explanation is like that given previously for Figure 9a.
(iv) With rising temperature, k_1,WM_ increases for both dyes (see Figure 11c). Again, the explanation is like the one given previously for Figure 9c, yet is further supported by results of Figure 11e, in which the corresponding B_1_ values drop.
(v) On the other hand, with increasing temperature k_2,WM_ decreases for both dyes (see Figure 11d), due to increasing S-L boundary layer thickness and film layer diffusion resistance (see B_2_ values in Figure 11f). The same trend is exhibited in Figure 9d.
(vi) The evolution of k_2,WM_ with growing dye load in the liquid phase (see Figure 11b) resembles that of k_2,DE_ (see Figure 9b), hence may be explained the same way.

The temperature-dependent rate coefficient values in Figure 11c,d enabled the calculation of apparent activation energies presented in Table 4. The faster stage generated values of 14.16 and 12.92 KJ/mole for MB and MBI, respectively. These are almost the same as their correspondents given by the DE model and suggest transport (diffusion)-controlled processes [35]. The WM slow stage also generated negative values: −22.87 for MB and −20.79 KJ/mole for MBI, respectively (similar to the DE model). Again, these negative apparent activation energies have no physical meaning; they merely reflect the extent of the temperature inhibition effect on the second slower process stage.

In conclusion, three rate laws describe best the adsorption’s advance in time for both methylene blue and its analog dye. These are the pseudo-second-order and the double-exponential empirical models, as well as the intraparticle diffusion Weber–Morris model in a double-linear approach. All were characterized by good values of model quality statistical indicators. Furthermore, both PSO and DE generated calculated equilibrium adsorption capacity values comparable with the experimental ones. The findings of DE and WM bi-linear models are similar. They both describe the process advance as a succession of two individual diffusion-controlled stages. The first is attributed to a faster, but mainly intraparticle-controlled step, in this case inside the interstitial spaces of the adsorbent. The second stage is slower and controlled mainly by S-L film diffusion.

These conclusions agree with those published previously for MB. Its adsorption kinetics was often described in the literature as obeying a pseudo-second-order rate law [6,10,28,35,36,37,38,39], or being diffusion-controlled [14,29,39,40,41], after checking at least three kinetic models. On the other hand, information regarding 3,7-bis(N,N-(2-hydroxyethyl)amino)-phenothiazinium iodide is reported here in premiere.

### 2.5. Equilibrium Data and Models

#### 2.5.1. Equilibrium Data

**Figure 12:**
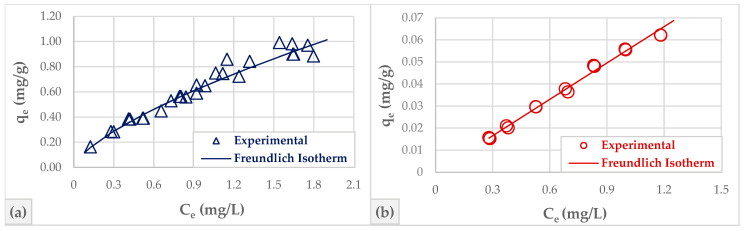
Experimental data and Freundlich isotherm fitted curves for equilibrium q_e_ vs. C_e_ plots, at 298 K, for (**a**) MB and (**b**) MBI.

Adsorption may be either chemical, physical, or a combination of both. Information related to these aspects is given by equilibrium data gathered at constant temperature, such as those presented in Figure 12. It illustrates experimental values of equilibrium adsorption capacity q_e_, the solid retained dye at 24 h after process initiation, plotted against the corresponding remanent dye concentration in the liquid bulk C_e_, by means of triangles and circles for MB and MBI, respectively.

**Figure 13:**
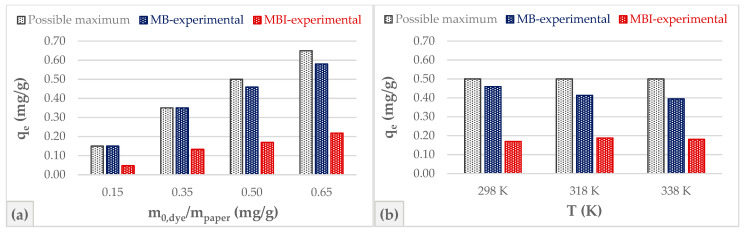
Average equilibrium adsorption capacity q_e_ values as compared to the 100% adsorption maximum value, as a function of the following: (**a**) m_0,dye_/m_paper_, at 298 K; (**b**) temperature, at m_0,dye_/m_paper_ of 0.5 mg/g.

The plots capture 298 K adsorption isotherms at low C_e_ values and hence are almost linear, with a slight tendency to level off towards higher concentrations. Plots obtained at 318 and 338 K, respectively, were alike and almost superposed the one at 298 K (within the limits of experimental errors). This fact is reflected also in the findings and corresponding discussions of Figure 13b and Section 2.5.3, respectively.

The adsorbent exhibits lower adsorption capacity towards MBI than MB, since q_e_ values in Figure 12b are approximately one order of magnitude smaller than the corresponding ones in Figure 12a. This is probably due to steric impediments (repulsion) faced by MBI when in the vicinity of the adsorbent’s cellulose structure (see Section 2.5.3).

Figure 13 also sustains this finding. It compares, for both MB and MBI, the potential maximum q_e_ value (meaning 100% adsorption), with its average experimental correspondent, at different m_0,dye_/m_paper_ (Figure 13a) and temperature (Figure 13b), respectively. MB was completely removed from its aqueous solution at low m_0,dye_/m_paper_ ratio values, because it was retained by both the paper and the inner surface of employed glass recipients (see Figure 2b and Figure 3). As m_0,dye_/m_paper_ increased, more MB was retained by paper and less by glass. Thus, q_e_ became closer to its possible maximum value. This was not the case for MBI. Although not retained by glass, and showing slightly higher q_e_ values with increasing m_0,dye_/m_paper_, it reaches less than half of the corresponding MB values.

Figure 13b proves that increased temperature has little—for MBI, and a rather negative effect—for MB, on their respective adsorption equilibria. This suggests a slightly exotherm physisorption for both dyes.

#### 2.5.2. Employed Adsorption Isotherm Models and Choice of Most Suitable Model

Various adsorption isotherms were reported for the removal of methylene blue from aqueous solutions, such as Langmuir [14,35,37,38,39,40,41,42], Freundlich [6,41,42], Sips [10,11], Redlich–Peterson [12], and Radke–Prausnitz [43], yet Temkin [11,14,36], Toth [10], and many others [44] were also tested.

Therefore, Table 5 summarizes the 11 adsorption isotherm models [29,45,46] chosen to fit 298 K equilibrium data from Figure 12a,b, respectively. In each case, the parameters calculated by means of curve fitting are listed for both MB and MBI, together with the values of model quality indicators R^2^_adj_ and Hybrid (see Section 2.3 and Appendix A). The isotherms were chosen to reflect a large range of assumptions for adsorption mechanisms, as well as by taking into consideration previously reported findings for methylene blue. The best-suiting isotherm was further applied for data gathered at other temperatures.

The first in Table 5 is the simplest, linear Henry model. It was chosen because it suits data at relatively low adsorbate concentrations (like those in Figure 12) [44]. The next two isotherms are the most often reported ones. The Langmuir model considers homogeneous monolayer chemical sorption, whereas Freundlich is an empirical isotherm, which corrects the Henry’s equation by introducing an adsorbent surface heterogeneity related coefficient [44,46]. It might also involve multilayer adsorption. Models 4 to 9 are some combination of models 2 and 3. They can be reduced under certain conditions either to isotherm 2 or 3, like Redlich–Peterson and Sips, or just to Equation (2), like the Toth model. The latter also takes into consideration the adsorbent’s surface heterogeneity. Kahn’s isotherm was reported most suitable for bi-adsorbate adsorption from pure dilute aqueous solutions [44], whereas the Hill isotherm considers that adsorbed molecules influence the adsorbent’s other binding sites [46]. The Radke–Prausnitz model is suited for large adsorbate concentration ranges [44]. It becomes linear at low values but converts into the Freundlich equation at higher ones. On the other hand, the Temkin empirical isotherm is valid for mid-range adsorbate concentrations and considers that the adsorption heat of individual molecules is affected by the adsorbent’s surface coverage [45,46]. The last model in Table 5 is based on the Polanyi’s potential theory [44,45,46].

The best-fitting model in Table 5 was chosen so that, simultaneously, R^2^_adj_ values closest to 1 and Hybrid values closest to 0 were met for both dyes. Because data for MBI follow a less scattered and almost linear trend (see Figure 12b), they fitted almost every isotherm well, apart from the equations of isotherms (4), (7), and (11) (see Table 5). But the ones which best satisfied the model quality statistical criteria for both dyes were the Henry and the Freundlich isotherms. The first may be a good fit for the employed relatively narrow range of low adsorbate concentrations, yet it does not generate a zero intercept as it ought to according to its equation in Table 5 (most certainly not for MB—see Figure 12a). Hence, the choice fell on model 3, a corrected model 1 by means of the adsorbent’s surface heterogeneity exponent 1/n.

#### 2.5.3. Adsorption Isotherm, Thermodynamic Parameters, and Adsorption Mechanism

The suitability of the Freundlich isotherm is also proven by its good superposition over experimental points shown in Figure 12a,b, for MB and MBI, respectively—see the continuous curves. Its parameters are presented in Table 6, for all employed temperatures. The values of C_e_ and q_e_ have been uniformized to mg/L for the calculus of the adsorption constants K_F_ [6,40].

The Freundlich model assumes multilayer heterogeneous adsorption. The value of K_F_ is considered an indicator of the adsorbate’s affinity towards the adsorbent; higher values are desired for favorable adsorption. The same is true for the dimensionless empirical exponent 1/n, if it lays between 0.1 and 1 [6,10,40].

The commercial filter paper used in this study has not been developed for the purpose of adsorption but rather for the opposite, to let liquid dissolved species through its net of cellulose fibers. Hence, the relatively small K_F_ values for MB and the one order of magnitude smaller ones for MBI. The literature mentions at least one order of magnitude higher K_F_ values for MB [6,10,12,40,42], but also comparable ones with those listed in Table 6, for some raw date-palm seeds [14] (also containing cellulose), pristine baker’s yeast [36] and *Leonurus cardiaca* L. biomass powder [11], respectively. The 1/n values in the 0.1 to 1 range for MB support the finding that its adsorption is favored. The sub-unitary K_F_ values and the close to 1 values of 1/n for MBI explain its weak affinity to the employed paper, hence the far smaller solid retained quantities than in the case of MB under similar experimental conditions.

(4) $$ln\left(K_{F}\right)=\frac{-∆G^{0}}{RT}=\frac{∆S^{0}}{R}-\frac{∆H^{0}}{R}\times \frac{1}{T}$$

The standard Gibbs energy variation ΔG^0^ was calculated by means of Equation (4), whereas standard enthalpy ΔH^0^ and entropy ΔS^0^ changes were determined from the plot of ΔG^0^ vs. the reverse of temperature. The results are summarized in Table 7. Values of ΔG^0^ for MB, within the −20 and 0 KJ/mole range, show that its adsorption is spontaneous and due to physisorption [7,11,13,14,35,39]. The ΔG^0^ for MB values in Table 7 agree with data reported previously for sulphated and other nanocellulose surfaces [8,42] as well as for pine-cone-derived biomass [35]. The slight decrease of K_F_ at higher temperature was also reported by Batmaz et al. [42]. It is responsible for the slight adsorption inhibition at 318 and 338 K, as compared to 298 K (see Figure 13b). On the other hand, the close to zero and slight positive ΔG^0^ for MBI is due to its very poor adsorption.

Scheme 1c illustrates the linear polysaccharide structure of *α*-cellulose, the main constituent of the employed adsorbent [9]. Each repeating unit contains one methylol and two hydroxyl polar functional groups, making the chain slightly negatively charged. Hokkanen et al. [7] state that adsorption on cellulose is mainly due to physical forces, such as Van der Waals forces, hydrogen bonds, polarity, and steric interactions, as well as others. Thus, in the case of physisorption methylene blue accumulates on the adsorbent’s surface by means of the above-listed interactions. On the other hand, the MBI molecule is larger than that of MB and contains four ethylol groups. Because of their polarity and slightly negative charge, they are sterically impeded to bond to the cellulose chain, thus adsorbing more poorly.

Due to the negative ΔH^0^ values in Table 7, it is concluded that both processes are slightly exothermic, the one for MB more so. The ΔH^0^ of MB is comparable with the one reported for cellulose nanocrystal-alginate hydrogel beads [39] and pine-cone biomass [35]. Moreover, the review of Lombardo et al. [8] mentions some other cellulose-based adsorbents which retain MB by means of exotherm processes. Exothermicity also sustains/explains the adsorption inhibition at higher temperatures.

For both dyes, ΔS^0^ is negative over the employed 40 degrees temperature range, suggesting less randomness at the adsorbent’s surface at equilibrium [11,14,39]. For MB, the value agrees with a previously reported one [39]. The small negative ΔS^0^ for MBI indicates that its adsorption induces insignificant changes both in the water molecules (solvent) arrangement at the S-L interface and in the inner structure of the adsorbent [35].

## 3. Materials and Methods

Commercially available methylene blue (MB), with a 319.85 g/mole molecular mass, provided by Merck KgaA, Darmstadt, Germany (CAS Number 122965-43-9) was used to prepare an aqueous 500 mg/L stock solution. This was further employed to freshly prepare, before each set of experiments, another stock of 25 mg/L concentration. The latter served the preparation of 1 to 15 mg/L working solutions, used in calibration (664 nm absorbance vs. MB concentration), kinetic, and equilibrium experiments, respectively. The 3,7-bis(N,N-(2-hydroxyethyl)amino)-phenothiazinium dye (MBI) was synthetized, purified, and characterized prior to this study, within the present research group, by a procedure described in the literature [12]. Its molecular mass is of 531.41 g/mole. Stock and working solutions were prepared like MB. All aqueous solutions were obtained by using deionized (HydroPure 300, MultiLab, Bucharest, Romania) and double distilled water (Lauda Puridest PD-2D, MultiLab, Bucharest, Romania).

Calibration lines A_664_ vs. dye concentrations were obtained with data corresponding to 8 different C_0,dye_ values in the range of 0.5 to 7 mg/L, for both MB and MBI, respectively.

The employed solid adsorbent was a cellulose-based, commercially available, standardized, white, creped, medium speed filter paper for general laboratory applications, marked as product MN 751/60, Lot CB048011 (Macherey-Nagel GmbH, Düren, Germany) [9]. The sheets are 0.27 mm thick and have a basis weight of 75 g/m^2^. It was used as provided by the manufacturer but cut in the shape of round flakes of 5 mm diameter.

Batch adsorption experiments were carried out in 8 mL borosilicate glass vials (Labbox Labware, Bucharest, Romania) with lids, for limiting the effect of water evaporation on the experimental data. Adsorption start (zero time) was operationally defined by the moment at which the paper flakes were rapidly placed into the vial containing 5 mL of dye solution. Each experiment employed the same amount of 0.05 g paper; hence, the initial adsorbate/adsorbent mass ratio was varied by varying the initial dye concentration of the aqueous solution. No stirring was employed, other than a short shaking of the vials after process initiation.

To monitor the advance of adsorption as a function of total S-L contact time, series of 17 to 24 identical water–dye–paper mixtures were prepared. At predetermined time intervals after adsorption initiation, 3.5 mL aliquots of the bulk solution were separated from the solid via extraction with a pipette. Their absorbance values were recorded at 664 nm, by using quartz cuvettes of 1 cm optical path length, and a Jasco V-650 UV-VIS spectrophotometer (ABL&E-JASCO România S.R.L., Cluj-Napoca, Romania). Adsorption equilibrium was completed at 24 h after process initiation. The remanent absorbance values of the bulk liquid were recorded as described above. Experiments were carried out at three different constant temperatures: 298, 318, and 338 K, respectively, ensured by means of a dry-heat oven with natural gravity convection (Ecocell ECO111, BMT Medizintechnik GmbH, Messkirch, Germany). Borosilicate volumetric flasks (Labbox Labware, Bucharest, Romania), MicroPette Plus one channel pipettes (Amex Healthcare GmbH, Viena, Austria) with adjustable volumes, and a Kern ABT220-5DNM analytical scale (SC. Driatheli Group SRL, Kern Official Partner, Ghiroda, Romania) were also used in experiments.

The scanning electron microscope images of the adsorbent were recorded by means of a Hitachi SU 8230 (Hitachi Industrial Products Ltd., Hitachi City, Japan) equipment, set at an acceleration voltage of 30 kV, resolutions between 10 μm and 1 mm, and penetration depths of 10 to 15 μm, respectively. The adsorbent’s BET surface was determined by means of a Micromeritics Flex & 3Flex (Micromeritics GmbH, Unterschleissheim, Germany), with N_2_ gas adsorption at 77.1 K.

Each experiment was carried out in triplicate, with relative standard deviation RSD values (standard deviation divided by average) between 0.32 and 3.78%. Necessary calculus was carried out by using Origin Pro 2018 v9.5.1 and the EXCEL media of Microsoft 365 and Office Updates, Version 2403.

## 4. Conclusions

A comparative adsorption study was carried out for methylene blue (MB) and its 3,7-bis(N,N-(2-hydroxyethyl)amino)-phenothiazinium dye analog (MBI). S-L batch experiments were carried out by using aqueous MB and MBI solutions of various initial concentrations and the same amount of commercial filter paper. In all employed conditions, more MB was retained than MBI.

Time-resolved experimental data were tested against seven kinetic models, and the decision upon the best one was aided by statistical quality indicators R^2^_adj_, RMSE, and Hybrid. Additionally, error distribution diagrams helped the ruling out of models with unproper trends. Although the Elovich rate law was not completely ruled out, the best suitable kinetic models were the pseudo-second-order, the double-exponential, and the bi-linear Weber–Morris equations. The first two also generated equilibrium adsorption capacities comparable to experimental values.

For both dyes, the process was proven to follow a succession of two diffusion-controlled steps. The first stage occurs after initiation, is faster, and is mainly controlled by external and internal diffusion (inside the interstitial spaces of adsorbent). The second step is slower, describes the process closer to equilibrium, and is mainly controlled by the S-L boundary’s film diffusion. Both the double-exponential and the bi-linear Weber–Morris models sustained the above finding; they also generated similar activation energy values. The rapid step is temperature-enhanced, whereas the slower one is inhibited.

Equilibrium data were tested against 11 isotherms chosen to correspond to the most often reported types for MB, as well as to reflect a large variety of adsorption mechanism assumptions. R^2^_adj_ and Hybrid statistical parameters were used to assess their suitability. The Freundlich isotherm described best experimental data for both MB and MBI. Calculated isotherm parameters together with Gibbs free enthalpy values suggested that in both cases physisorption is responsible for the dye removal from its aqueous solution. The adsorption of MB is thermodynamically favored, whereas that of MBI is not. Both processes proved to be slightly exothermic, thus being somewhat inhibited at higher temperatures. Both induced less degrees of randomness at the adsorbent’s surface at equilibrium.

Finally, the employed filter paper is suitable for retaining MB from its aqueous solutions, since this dye’s adsorption is spontaneous, thermodynamically favored, yet exothermic. Because of the latter, room or lower temperatures are recommended for better performance. On the other hand, this filter paper ought to be used rather for filtration/purification purposes of MBI aqueous solutions. It retains some MBI, yet this dye’s adsorption is not thermodynamically favored. Since there are no literature data regarding the adsorption behavior of this methylene blue analog, the above-presented data are a premiere.

## Figures and Tables

**Table 1:** Employed adsorption kinetic models, their corresponding rate laws, and kinetic parameters.

Kinetic Model	Rate Law ^4^	Kinetic Parameters	Plot Coordinates
	2-parameter models		
PFO ^1,3^	$ln\left(q_{e}-q\right)=ln\left(q_{e}\right)-k_{PFO}t$	-Rate coefficient k_PFO_ (1/min) -Calculated q_e_ (mg/g)	$ln\left(q_{e}-q\right)\;vs.\;t$
PSO ^1,3^	$\frac{t}{q}=\frac{1}{k_{PSO}{\left(q_{e}\right)}^{2}}+\frac{1}{q_{e}}t$	-Rate coefficient k_PSO_ (g/mg·min) -Calculated q_e_ (mg/g)	$\frac{t}{q}\;vs.\;t$
Avrami ^1,3^	$ln\left(ln\frac{q_{e}}{q_{e}-q}\right)=nln\left(k_{Avrami}\right)+nln\left(t\right)$	-Rate coefficient k_Avrami_ (1/min^n^) -Model constant n (dimensionless)	$ln\left(ln\frac{q_{e}}{q_{e}-q}\right)\;vs.\;ln\left(t\right)$
Vermeulen ^2,3^	$ln\left[1-{\left(\frac{q}{q_{e}}\right)}^{2}\right]=k_{Vermeulen}t$	-Intraparticle diffusion rate coefficient k_Vermeulen_ (1/min). -Intercept (dimensionless)	$ln\left[1-{\left(\frac{q}{q_{e}}\right)}^{2}\right]\;vs.\;t$
Weber–Morris ^2,3^	$q=k_{WM}\sqrt{t}+B$	-Intraparticle diffusion rate coefficient k_WM_ (mg/g·min^0.5^) -Model constant B (mg/g)	$q\;vs.\;\sqrt{t}$
Elovich ^2,3^	$q=\frac{1}{\beta }ln\left(\alpha \beta \right)+\frac{1}{\beta }ln\left(t\right)$	-Initial adsorption rate α (mg/g·min) -Model constant β (g/mg)	$q\;vs.\;ln\left(t\right)$
	4-parameter model		
DE ^1,2^	$\left(q_{e}-q\right)=A_{1}\exp\left(-k_{1,DE}t\right)+A_{2}\exp\left(-k_{2,DE}t\right)$	-Rate coefficient of rapid first step at process initiation k_1,DE_ (1/min) -Rate coefficient of slow second, near equilibrium, step k_2,DE_ (1/min) -Model constants A_1_, A_2_ (mg/g)	$\left(q_{e}-q\right)\;vs.\;t$

^1^ Empirical kinetic model. ^2^ Adsorption mechanism describing kinetic model. ^3^ Linearized expression of rate law. ^4^ In all rate laws, t (min) stands for a certain moment in the process evolution, q (mg/g) for its corresponding paper adsorption capacity value, whereas q_e_ (mg/g) stands for the equilibrium value of q, under certain experimental conditions.

**Table 2:** Average values for kinetic parameters of the PSO and Elovich rates laws, for both MB and MBI, under various experimental conditions.

		PSO Rate Law		Elovich Rate Law			
		k_PSO_ (g/mg·min)		α (mg/g·min)		β (g/mg)	
T (K)	m_0,dye_/m_paper_ (mg/g)	MB	MBI	MB	MBI	MB	MBI
298	0.35	0.140	0.434	0.094	1.021	18.87	57.80
	0.50	0.075	0.236	0.050	1.042	13.51	44.82
	0.65	0.062	no data	0.063	1.046	11.05	31.06
318	0.50	0.139	0.441	0.077	1.081	12.81	37.88
338	0.50	0.328	0.782	0.152	1.114	14.43	40.49

**Table 3:** Bi-linear Weber–Morris kinetic model constant B values for m_0,dye_/m_paper_ of 5.0 mg/g and 298 K.

WB Model Constant B	MB	MBI
B_1_ (mg/g)	0.036 ± 0.007	0.041 ± 0.009
B_2_ (mg/g)	0.211 ± 0.068	0.069 ± 0.005

**Table 4:** Apparent activation energy values for process stages identified by means of various kinetic models, and based on rate coefficient vs. temperature data enabled by the respective rate laws, at m_0,dye_/m_paper_ of 0.5 mg/g.

Kinetic Model	Adsorption Mechanism/Rate-Controlling Stage	Apparent Activation Energy (KJ/mole)	
		MB	MBI
PSO	Overall process/Not specified	30.74 ± 3.89 (R^2^_adj_ = 0.9661)	25.12 ± 0.28 (R^2^_adj_ = 0.9999)
DE	Rapid debut stage/External and internal diffusion	16.32 ± 7.53 (R^2^_adj_ = 0.8342)	16.36 ± 1.71 (R^2^_adj_ = 0.9799)
	Slow stage near equilibrium/Internal diffusion	(−22.27 ± 7.68) * (R^2^_adj_ = 0.8000)	(−41.91 ± 9.18) * (R^2^_adj_ = 0.9144)
WM bi-linear	Rapid debut stage/Interstitial space diffusion control (stands for intraparticle diffusion)	14.16 ± 0.69 (R^2^_adj_ = 0.9956)	12.92 ± 0.15 (R^2^_adj_ = 0.9997)
	Slow stage near equilibrium/S-L boundary layer film diffusion control	(−22.87 ± 0.22) * (R^2^_adj_ = 0.9998)	(−20.79 ± 3.36) * (R^2^_adj_ = 0.9524)

* These values have no physical meaning; they merely express the extent of the rate’s temperature dependence.

**Table 5:** Equations, parameters, and model quality statistic parameters for the tested adsorption isotherm models, at 298 K, for both MB and MBI.

Isotherm Model	Equation and Model Parameters ^4,5^	Parameters ^4^		R^2^_adj_ & Hybrid	
		MB	MBI	MB	MBI
1. Henry ^1^	$q_{e}=K_{HN}C_{e}$	K_HN_ = 0.475	K_HN_ = 0.054	0.9468 1.0573	0.9911 0.1650
2. Langmuir ^2^	$q_{e}=\frac{q_{sL}K_{L}C_{e}}{1+K_{L}C_{e}}$	K_L_ = 2.308 q_SL_ = 0.850	K_L_ = 0.048 q_SL_ = 1.197	0.9540 5.130	0.9870 0.1885
3. Freundlich ^2^	$q_{e}=K_{F}{C_{e}}^{\frac{1}{n}}$	K_F_ = 0.665 1/n = 0.679	K_F_ = 0.055 1/n = 0.962	0.9621 2.7976	0.9942 0.1584
4. Redlich–Peterson ^3^	$q_{e}=\frac{K_{R}C_{e}}{1+q_{sR}{C_{e}}^{\beta_{R}}}$	K_R_ = 6143.6 q_sR_ = 9603.3 β_R_ = 0.3732	no fit for 0 < β_R_ < 1	0.7759 22.8531	No data
5. Sips ^3^	$q_{e}=\frac{q_{sS}K_{S}{C_{e}}^{n_{S}}}{1+K_{S}{C_{e}}^{n_{S}}}$	K_S_ = 0.0051 q_sS_ = 129.85 n_S_ = 0.7240	K_S_ = 0.528 q_sS_ = 0.178 n_S_ = 1.258	0.9199 6.6168	0.9939 0.1473
6. Toth ^3^	$q_{e}=\frac{q_{sT}C_{e}}{{\left(K_{T}+{C_{e}}^{t}\right)}^{\frac{1}{t}}}$	K_T_ = 2.3160 q_sT_ = 1.26 × 10^−14^ t = 0.2824	K_T_ = 9.0780 q_sT_ = 0.7093 t = 0.7751	0.9200 2.9978	0.9907 0.3007
7. Kahn ^3^	$q_{e}=\frac{q_{sK}K_{K}C_{e}}{{\left(1+K_{K}C_{e}\right)}^{n_{K}}}$	K_K_ = 367.63 q_sK_ = 0.0093 n_K_ = 0.2807	K_K_ = 0.0102 q_sK_ = 0.5722 n_K_ = 0.7021	0.9199 3.0198	0.9911 112.00
8. Radke–Prausnitz ^3^	$q_{e}=\frac{q_{sRP}K_{RP}C_{e}}{1+K_{RP}C_{e}^{\beta_{RP}}}$	K_RP_ = 1.06 × 10^20^ q_sRP_ = 0.650 β_RP_ = 0.2963	K_RP_ = 0.1389 q_sRP_ = 0.6012 β_RP_ = 0.4454	0.9087 2.8878	0.9905 0.2903
9. Hill ^3^	$q_{e}=\frac{q_{sH}{C_{e}}^{n_{H}}}{K_{H}+{C_{e}}^{n_{H}}}$	K_H_ = 77,040 q_sH_ = 50,023 n_H_ = 0.7038	K_H_ = 1.9309 q_sH_ = 0.1608 n_H_ = 1.2890	0.9087 2.8878	0.9934 0.1676
10. Temkin ^2^	$q_{e}=\frac{RT}{b}ln(K_{Tm}C_{e})$	K_Tm_ = 6.883 b = 7197.7	K_Tm_ = 5.015 b = 73,923.6	0.7871 22.920	0.9713 1.6214
11. Dubinin–Radushkevich ^2^	$q_{e}=q_{sDR}e^{-\left(K_{DR}\varepsilon^{2}\right)}$ $\varepsilon =RT\left(1+\frac{1}{C_{e}}\right)$	K_DR_ = 5.02 × 10^−8^ q_sDR_ = 0.740	K_DR_ = 1.19 × 10^−7^ q_sDR_ = 0.075	0.7404 64.130	0.9694 118.58

^1^ One-parameter adsorption isotherm. Model ^2^ Two-parameter adsorption isotherm model. ^3^ Three-parameter adsorption isotherm model. ^4^ Values of C_e_ and q_e_ expressed in mg/L and mg/g, respectively. ^5^ Signification of parameters: q_sL_, q_sR_, q_sS_, q_sT_, q_sK_, q_sRP_, q_sH_, q_sDR_—maximum absorption capacities for models 2, 4, 5, 6, 7, 8, 9, and 11, respectively; K_HN_, K_L_, K_F_, K_R_, K_S_, K_T_, K_K_, K_RP_, K_H_, K_Tm_, K_DR_—equilibrium constants of models 1 to 11, respectively; 1/n—surface heterogeneity coefficient (0 < 1/n < 1); β_R_—Redlich–Peterson exponent, (0 < β_R_ < 1); n_S_—Sips isotherm exponent, one adsorbate molecule occupies 1/n_S_ binding sites; t—Toth constant related to heterogeneity, (0 < t < 1); n_K_—Kahn isotherm exponent; β_RP_—Radke–Prausnitz isotherm exponent; n_H_—Hill’s affinity coefficient describing binding interaction [32], b—Temkin isotherm constant, related to adsorption heat; R—universal gas constant (8.314 J/mole·K); T—temperature (K); ε—Polanyi’s adsorption potential.

**Table 6:** Freundlich isotherm parameter values for both MB and MBI.

T (K)	K_F_ (mg/L)^(1−1/n)^		1/n (Dimensionless)		R^2^_adj_	
	MB	MBI	MB	MBI	MB	MBI
298	6.54 ± 0.28	(5.50 ± 0.03) × 10^−1^	0.679 ± 0.025	0.962 ± 0.020	0.9621	0.9947
318	3.40 ± 0.09	(4.37 ± 0.06) × 10^−1^	0.759 ± 0.031	0.831 ± 0.061	0.9772	0.9687
338	3.37 ± 0.13	(4.86 ± 0.06) × 10^−1^	0.603 ± 0.043	0.971 ± 0.065	0.9331	0.9199

**Table 7:** Thermodynamic parameter values for both MB and MBI.

T (K)	ΔG^0^ (KJ/mole)		ΔH^0^ (KJ/mole)		ΔS^0^ (J/mole·K)	
	MB	MBI	MB	MBI	MB	MBI
298	−4.65	1.48				
318	−3.24	2.19	−14.16	−2.74	−32.68	−14.59
338	−3.41	2.03				

## Data Availability

The data presented in this study are available upon request from the corresponding author.

## Appendix A

The statistical parameters employed in the kinetic and isotherm model quality assessment are described and argued for below:

- The adjusted coefficient of determination (R^2^_adj_) is one of the most often used such indicators [27]. Since it correlates the R^2^ determination coefficient with the number of model parameters (N_para_), as well as the amount of data employed in their calculus (N_exp_)—see Equation (A1), it enables fair comparison both between models with different N_para_ and experiments with unequal N_exp_. Values close to 1 are ideal, yet models with R^2^_adj_ above 0.9 are considered acceptable.

(A1) $$R_{adj}^{2}=1-\left(1-R^{2}\right)\frac{\left(N_{exp}-1\right)}{\left(N_{exp}-N_{para}-1\right)}$$

- Another widely used parameter is the root means square error (RMSE), also called root means square deviation (RMSD) [26,28]. Its use tests the superposition between the model predicted/calculated and the experimental values—see Equation (A2). Since it takes into consideration only N_exp_, it may be used to compare various models having equal N_para_. Very good models have close to zero RMSE values.
  (A2) $$RMSE=\sqrt{\frac{\sum_{i=1}^{N_{exp}}{\left(q_{exp}-q_{calc}\right)}_{i}^{2}}{N_{exp}}}$$
  q_exp_ and q_calc_ stand for the experimental and model calculated values, respectively, of adsorption capacity q, for the data point “i”.

- To overcome the shortcomings of RMSE, which assumes that errors are unbiased, free from outliers, and follow normal distribution [26], some researchers used another error evaluation function, which balances absolute deviations against fractional error [26]. The hybrid fractional error deviation (Hybrid), described by Equation (A3), considers both N_exp_ and N_para_. Hence, it is more suitable than RMSE for comparison of models with unequal N_para_. Very good models have close to zero Hybrid values.

(A3)
$$Hybrid=\frac{100}{N_{exp}-N_{para}}\sum_{i=1}^{N_{exp}}{\left[\frac{{\left(q_{exp}-q_{calc}\right)}^{2}}{q_{exp}}\right]}_{i}$$

- Another method of assessing whether a certain model fits the entire experimental range of data, for example, in kinetic measurements of the entire evolution in time of the process, is the plot of error distribution of residuals as related to the corresponding mean experimental value—see Equation (A4), against/within the considered experimental range.
  (A4) $${Error}_{i}=100{\left(\frac{q_{exp}-q_{calc}}{q_{exp}}\right)}_{i}(\%)$$
  It helps identifying undesired trends of just positive or negative, as well as continuously increasing or decreasing error series. Hence, symmetric distribution around zero value is desired. It must be mentioned that model error distribution diagrams ought to be used for the assessment of model prediction quality only if the errors in recording experimental data (values of q_exp_) do not exceed more than ±10% [26].

## Appendix B

**Table A1:** Kinetic model quality indicators R^2^_adj_, RMSE, and Hybrid for MB adsorption under various experimental conditions and according to the rate laws of the following: (**a**) PFO; (**b**) PSO; (**c**) Avrami; (**d**) Vermeulen; (**e**) Weber–Morris; (**f**) Elovich; (**g**) DE models, respectively.

		**(a) PFO**			**(b) PSO**		
**T (K)**	**m_0,dye_/m_paper_ (mg/g)**	**R^2^ _adj_**	**RMSE**	**Hybrid**	**R^2^ _adj_**	**RMSE**	**Hybrid**
298	0.15	0.9375	0.0462	27.139	0.9996	0.0099	1.577
	0.35	0.9059	0.0905	36.244	0.9985	0.0245	4.559
	0.50	0.9546	0.0952	35.992	0.9871	0.0362	4.900
	0.65	0.9768	0.0882	29.093	0.9432	0.0551	7.875
318	0.50	0.9758	0.0640	16.973	0.9961	0.0208	2.326
338	0.50	0.8807	0.1013	24.539	0.9976	0.0181	1.059
		**(c) Avrami**			**(d) Vermeulen**		
**T (K)**	**m_0,dye_/m_paper_ (mg/g)**	**R^2^ _adj_**	**RMSE**	**Hybrid**	**R^2^ _adj_**	**RMSE**	**Hybrid**
298	0.15	0.9141	0.0501	24.238	0.9212	0.0203	5.490
	0.35	0.9708	0.1627	72.775	0.9338	0.0310	4.970
	0.50	0.9627	0.2345	100.063	0.9749	0.0363	5.033
	0.65	0.9317	0.2411	97.948	0.9456	0.0485	6.529
318	0.50	0.9567	0.1373	30.657	0.9575	0.0212	2.008
338	0.50	0.9191	0.1078	69.177	0.9291	0.0314	1.840
		**(e) WM mono-linear**			**(f) Elovich**		
**T (K)**	**m_0,dye_/m_paper_ (mg/g)**	**R^2^ _adj_**	**RMSE**	**Hybrid**	**R^2^ _adj_**	**RMSE**	**Hybrid**
298	0.15	0.6729	0.0945	77.736	0.9288	0.0055	0.285
	0.35	0.8029	0.1463	57.292	0.9706	0.0129	0.509
	0.50	0.9395	0.1054	25.543	0.9323	0.0196	1.144
	0.65	0.9889	0.0671	12.309	0.8871	0.0345	2.083
318	0.50	0.8797	0.1067	22.074	0.9707	0.0182	2.176
338	0.50	0.6978	0.1654	39.431	0.9206	0.0267	2.403
		**(g) DE**					
**T (K)**	**m_0,dye_/m_paper_ (mg/g)**	0.9538	0.0367	11.005			
298	0.15	0.9799	0.0670	11.461			
	0.35	0.9765	0.0226	0.8578			
	0.50	0.9745	0.0039	0.0242			
	0.65	0.9894	0.0282	1.1906			
318	0.50	0.9918	0.0553	4.0473			
338	0.50	0.9538	0.0367	11.005			
